# A qualitative exploration of the experiences of peer leaders in an intervention to improve diabetes medication adherence in African Americans

**DOI:** 10.1186/s12889-023-15059-2

**Published:** 2023-01-20

**Authors:** Adati Tarfa, Jenna Nordin, Mattigan Mott, Martha Maurer, Olayinka Shiyanbola

**Affiliations:** University of Wisconsin School of Pharmacy, Madison, WI USA

**Keywords:** African American, Peer leader, Peer support, Type 2 diabetes, Medication adherence

## Abstract

**Background:**

African Americans chronically managing their diabetes benefit from receiving support from peers with shared experiences. Peer support is known to improve the well-being of individuals receiving support, however, there is limited literature on the experiences of those providing the support. The Peers Supporting Health Literacy, Self-efficacy, Self-Advocacy, and Adherence (Peers LEAD) program pairs Peer Ambassadors who are adherent to their diabetes medication, with Peer Buddies who need support with their medication adherence. Peer Ambassadors engage with Peer Buddies as they receive diabetes information, develop the skills and motivation to address identified psychosocial/sociocultural issues to enhance their diabetes medication adherence. This study qualitatively explores the experiences of African Americans who provided peer support in the Peers LEAD medication adherence intervention.

**Methods:**

Two focus groups were conducted with twelve Peer Ambassadors to explore their experiences of providing peer support in the Peers LEAD medication adherence intervention. Qualitative content analysis was conducted using an inductive open coding approach.

**Results:**

Emergent themes provided insight into Peer Ambassador’ rationale for providing peer support and the benefits and challenges they experienced in their roles. Themes regarding their rationale included: their desire to receive support for their diabetes self-management as well as to contribute to their communities in reducing the stigma associated with diabetes. The perceived benefits they gained centered on creating interpersonal connections, experiencing personal growth as they adapted to their roles, and experiencing opportunities to contribute to an intervention regardless of professional training. Peer Ambassadors reflected on the challenges which included difficulties on coming to terms with their role as Peer Ambassadors, seeing African Americans experience complications associated with diabetes, and navigating supporting Peer Buddies who are also burdened with the challenges their family members are experiencing with managing their diabetes.

**Conclusions:**

This study provides unique insight to what motivates individuals to provide peer support and what they gain from these experiences despite the challenges. Understanding the experiences of peers participating in such interventions may help inform the structure and content of programs that use peer support to focus on the benefits of and the motivation for participating in the program.

## Introduction

An estimated 30.2 million people in the United States have diabetes, and the total annual cost associated with diagnosed diabetes is approximately $245 billion [1]. Chronic diseases such as diabetes remain a burdensome challenge to health care systems and treating people with chronic diseases accounts for 86% of health care costs [2, 3]. Diabetes-related costs alone is projected to reach $622.3 billion in 2030, including $472 billion in annual medical costs [1]. Thus addressing diabetes requires new strategies, to improve the quality of patients’ lives; increase access to healthcare services; and reduce healthcare costs by preventing or minimizing the effects of the illness [2]. However, healthcare systems are struggling to contain the increasing burden of diabetes, and the burden of disease management are increasingly falling on patients and their caregivers [4]. As such, diabetes self-management education and support are critical for diabetes management [5]. Diabetes self-management involves following a healthy diet, incorporating physical exercise into the daily routine, using medication correctly; monitoring blood glucose levels,, and making the right decisions regarding healthcare [6, 7]. A literature of self-management programs shows a majority are delivered by health professionals, however the use of peer support is more common and may be cost-effective [6].

Peer support interventions are cost-effective means of improving and sustaining the self-management of chronic diseases such as diabetes [8–10]. Peer supporters are individuals who may face similar self-management challenges and share the practical aspect of managing diabetes in day-to-day life [11]. The relationships between peers are established based on shared experiences and the challenges of living with a similar chronic health condition [12]. Through peer support interventions, participants receive social, emotional, and behavioral support while learning to cope with the demands of disease management. Peer support is beneficial for individuals with chronic diseases who are required to navigate and adhere to complex medication regimens [13]. This is especially true for individuals from marginalized populations or lower socioeconomic groups. For example, African Americans with type 2 diabetes experience unique sociocultural barriers (e.g., medical mistrust and distrust of medication) that contribute to poor diabetes medication adherence and health outcomes [14, 15]. Thus, connecting African Americans with individuals who identify with them and share common experiences may have greater credibility as a resource for achieving desirable health behaviors and advancing health equity [16]. Additionally, utilizing direct patient and community engagement to design peer support interventions is key to improving diabetes medication management in the African American community and reducing diabetes disparities [15]. A systematic review examining the effectiveness of peer support in adults with diabetes, reported that peer support groups should be culturally tailored to its target population [9].

Increasing attention is being given to racial and ethnic minority groups—including African-Americans--and development of diabetes self-management peer support programs that are culturally-adapted [17]. Peers Supporting Health Literacy, Self-Efficacy, Self-Advocacy, and Adherence (Peers LEAD) is a community-engaged intervention that provides culturally appropriate diabetes information to address the psychosocial/sociocultural barriers to diabetes medication adherence in African Americans with type 2 diabetes [18]. The Peers LEAD program partners African Americans, named “Peer Buddies”, with a “Peer Ambassador” who is considered the peer leader and is successfully managing their type 2 diabetes and taking their medicines. The experiences of peer leaders who provide diabetes support have been studied in a limited scope. Peer leader experiences have been captured in interventions among veterans [19] and African Americans with heart disease [16, 20]. Further investigating the experiences of peer leaders presents a unique opportunity to gain insights into the motivations, benefits, and barriers to serving in this capacity.

Peer support is shown to be beneficial for those who need to improve on their diabetes self-management, as well as for peer leaders who provide the support [13, 21]. Despite the advantages to peer leaders beneficial role in assisting patients with cancer screening and treatment [22, 23], diabetes control [20], and chronic cardiovascular disease [24] the literature on peer support interventions focus on the experiences of health care professionals and participants, with considerably less attention given to the peer leaders delivering the intervention [25]. Understanding the motivations, and challenges experienced by peer leaders may provide information on how to better design, recruit for, and provide peer support interventions, thereby potentially improving their uptake and effectiveness.

The objective of this paper is to qualitatively explore the experiences of African Americans who provided peer support in the Peers LEAD medication adherence intervention.

## Methods

### Overview of Peers LEAD

Details about the Peers LEAD intervention can be found in a protocol paper that describes the theoretical and conceptual frameworks that informed the study design, the participant recruitment/training procedure, and the intervention outcomes [26]. As such, the following intervention description is included to provide additional context to better understand the Peer Ambassador feedback. Peers LEAD is an 8-week educational-behavioral program that offers African Americans culturally informed diabetes and medication information, peer support from other African Americans with type 2 diabetes and the skills to promote self-efficacy and provider communication towards improving medication adherence. A key component of this intervention includes peer support and group education sessions, which were implemented to address psychosocial and sociocultural barriers to medication adherence that are unique to African Americans with type 2 diabetes. Subsequently, African Americans with type 2 diabetes who were adherent to their diabetes medications, known as Peer Ambassadors, were paired with African Americans with type 2 diabetes who were nonadherent to their diabetes medications, Peer Buddies [15].

### Recruitment of peer ambassadors

Purposeful sampling was used to recruit Peer Ambassadors. The research team asked community stakeholders and individuals who were Peer Ambassadors in previous studies to help identify Peer Ambassador candidates [15, 18]. The inclusion criteria for Peer Ambassadors were self-identifying as African American or Black, being between the ages of 30 and 65 years old, taking one or more oral diabetes medicine, being diagnosed with type 2 diabetes for at least 1 year, and the ability to communicate in English. Additionally, Peer Ambassadors had to be adherent to their diabetes medicines, which were assessed using the Adherence to Refills and Medication Scale—Diabetes (ARMS-D) [27]. To be eligible to be a Peer Ambassador, individuals needed to receive a score of 11 on the self-reported ARMS-D scale, indicating diabetes medication adherence.

Prospective Peer Ambassadors were screened via a phone conversation or face-to-face to assess their eligibility. During the screening, the research team assessed a candidate’s interest in serving on an advisory board, offering peer support, and their personal diabetes experiences.

Peer Ambassadors attested to completion of human subjects ethics training by signing an investigator responsibility form approved by the University’s Institutional Review Board and consented to participate in a focus group by reviewing a consent information sheet approved by the University’s Institutional Review Board.

### Peer ambassador training

The training of Peer Ambassadors was facilitated by the research team and consultants from the Wisconsin Network for Research Support (WINRS), a patient and community engagement group with experience in facilitating stakeholder engagement meetings and training since 2010 [18].

Peer Ambassadors attended an orientation session 3 weeks before the start of the eight-week intervention. The orientation lasted two-and-half hours. Peer Ambassadors were informed about the project goals and the roles of all project stakeholders. During the orientation, PAs also participated in a demonstration of a Peer Ambassador actively listening and providing support to their peer buddies. Then, 1 week before the eight-week intervention, Peer Ambassadors participated in a two-hour training session where they prepared for phone calls with Buddies, were provided with detailed phone call suggestions, and discussed a telephone call guide that the Peer Ambassadors adjusted to their conversation styles.

### Activities of peer ambassador in the Peers LEAD program

**Fig. 1:**
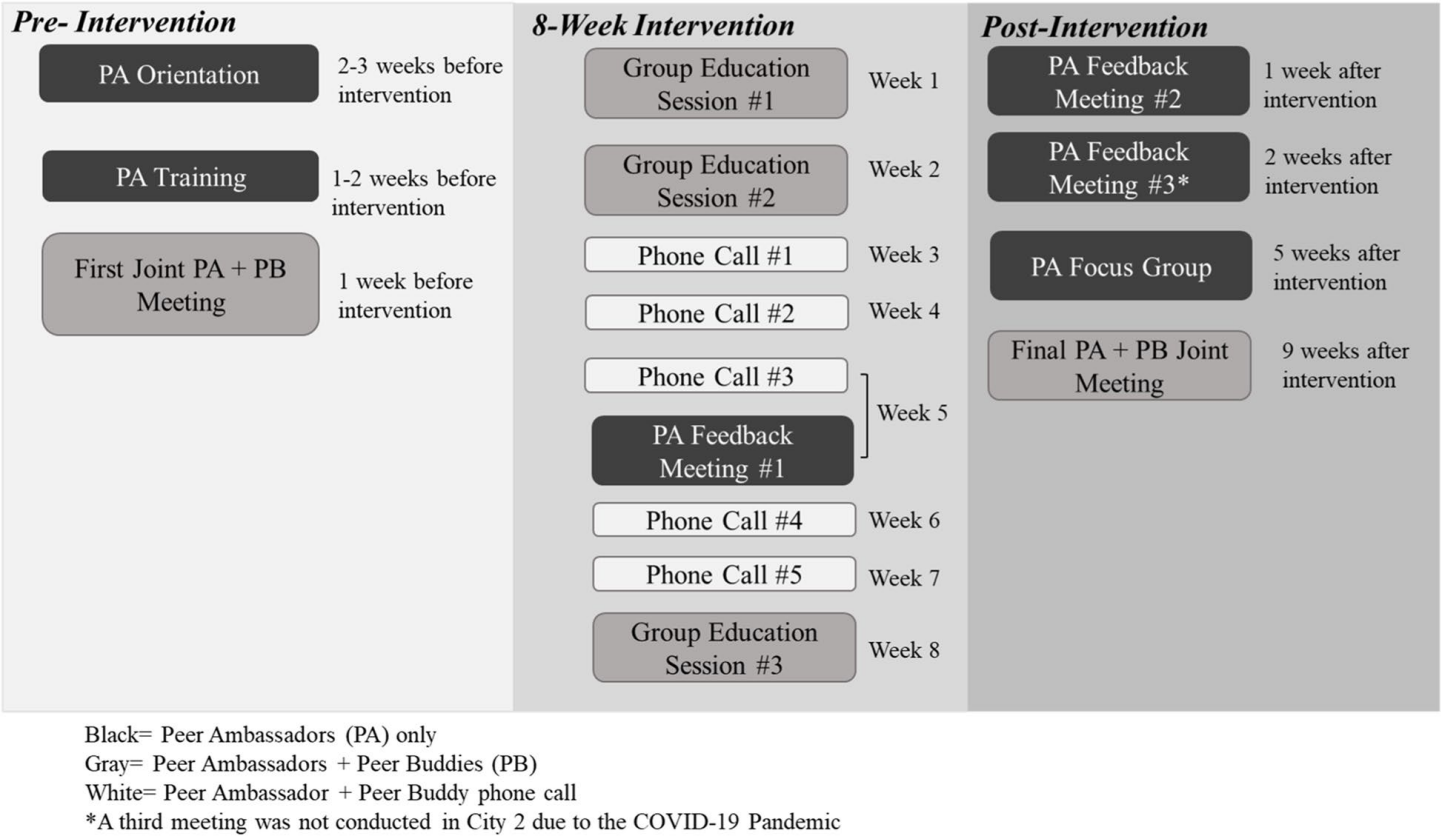
Activities for Peer Ambassador providing peer support in Peers LEAD diabetes self-management program

Fifteen peer ambassadors were paired with 21 Peer Buddies to provide support throughout the intervention. Peer Ambassadors were paired with one or two Buddies. Peer ambassadors attended 8 h of education with their peer buddies, including a separate Peer Ambassador/Buddy orientation and three group education sessions. Peer Ambassadors worked with their Buddies for 11 weeks, during which they attended the education sessions and completed five phone calls with their Peer Buddies (Fig. 1).

Figure 1 shows the activities that Peer Ambassadors participated in for the Peers LEAD Program.

## Data collection

### Focus group with peer ambassadors

All Peer Ambassadors who participated in the Peers LEAD program were invited to participate in a 90-minute focus group upon completion of the 8-week intervention. Compared to individual interviews, focus groups allow a range and depth of responses, and Peer Ambassadors can stimulate new thoughts for each other, which might otherwise not occur [26]. A focus group guide was developed by OS, MM, and WINRS (Table 1). The focus groups were facilitated by WINRS in two cities in a midwestern state. Peer Ambassadors were paid $50 each for participating in the focus group.

**Table 1:** Sample focus group questions for Peer Ambassadors

**Experience of being a Peer Ambassador**	
Tell us how you first became involved with Peers LEAD as a Peer Ambassador (PA)	
How would one describe the experience of being a peer ambassador to someone who is interested in doing this?	
Have PAs kept in touch with their Peer Buddies (PB) since the program ended?	
What was the most meaningful or valuable activity or time spent while participating in Peers LEAD as a PA?	
**Feedback on program benefits, challenges, and supports**	
What was beneficial about serving as a PA?	
What were the hardest parts about being a PA?	
What is the ongoing benefit or impact of Peers LEAD for the Peer Buddies?	
How well did the research team accomplish their goals?	
How did the research teamwork with PAs in a way that was meaningful, showing that their opinions matter?	
How does being a PA influence a PA’s behavior related to taking care of diabetes?	
**Advice on improving Peers LEAD**	
What is the best way to find interested potential PAs?	
What qualities are important for a peer ambassador to have?	
What could be done to improve the support from the research team for a PA?	
In what ways could PAs work with PBs be improved? What else could the research team do to improve: •The relationship between PAs and PBs •Activities during the program	
Are there things that the research team didn’t think about that would be important to address?	
Closing Question: Is there anything else that you would like to share with us about being a Peer Ambassador for the Peers LEAD?	

## Data analysis

All focus groups were audio-recorded and transcribed verbatim. Conventional content analysis [28] was used to organize themes. Research team members AT and JN initially read the data to achieve immersion. AT and NJ created codes, developed, and organized themes. A comparison of themes explored the similarities and differences. Any differences in coding were reviewed and resolved by the consensus of the research team. Subsequently, similar coded themes were sorted into categories, and similar responses in each category were grouped to find a description of the pattern. Ultimately, a detailed description of the Peer Ambassador experiences was generated. Thematic data saturation was reached when there were no new emerging ideas [29].

## Results

In total, 15 African Americans served as Peer Ambassadors in the Peers LEAD program. The mean age of the Peer Ambassadors was 57 (± 7.5) years. Of the Peer Ambassadors that were trained and participated in the Peers LEAD program, 12 consented to participate in the focus group.

When asked about their roles as Peer Ambassadors, three main topics emerged regarding the Peer Ambassador experiences: the benefits of being a Peer Ambassador, the challenges of being a Peer Ambassador, and the rationale for being a Peer Ambassador.

### The benefits of being a Peer Ambassador (PA)

Qualitative analysis of the focus groups revealed several personal benefits that the PAs attributed to serving in this role. Most PAs reported that serving as a PA offered an opportunity to make meaningful connections by opening up, building trust, and listening to their Buddies. The themes and subthemes are described below with verbatim quotes from the PAs. Table 2 shows other quotes used by the PAs to describe the benefits of performing in their role as PAs.

**Table 2:** Peer Ambassador’s perception of the benefits of being an ambassador

Themes	Sample quotes	
Creating interpersonal connections through opening up, trust-building, and listening	*“Well, mine [Peer Buddy] had a lot of medical problems and going through something, and I think it helped her a lot that I listened to that part, what she was going through medically… And then, as our conversation moved on, we was on the phone way longer than what we should have been. If she needed me to listen, I was there for her. That’s the part of her building that trust together with one another, just for me to be there to listen to other things as well as her diabetes.” – PA5*	
Opportunities for growth while being a PA	Subtheme 1: Opportunities to contribute regardless of professional training	*“I would have to say establishing new relationships. It was nice, and you know, although I was an ambassador that was a nurse, I didn’t have to be a nurse, you know. So, it was definitely friendship.”- PA11*
	Subtheme 2: Opportunities to grow and adapt to the PA role	*“And it not only helped me to give back, but it also helped me as well to be able to grow and to get better, as far as me doing the right thing.” – PA8*
	Subtheme 3: Opportunities for self-awareness and self-reflection on diabetes journey	*“And I learned a lot about myself, because people that don’t want to listen to me, then I typically don’t want to listen to them either. But I had to, in this case, because that’s what I was supposed to do.” – PA2* *“One way of describing it as being a positive mentor to others who have diabetes. And as you teach them, you also learn, so it’s an interchange of encouragement. And that’s what I would tell them. And I like that doctors, dieticians, pharmacists come to our session, so that we’re learning first how to benefit ourselves and then how to benefit the peer buddy.” – PA3*
Experience the reward that comes with the commitment	*“The experience of being a Peer Ambassador, it’s interesting. You are able to get close to people that are dealing with the same chronic disease that you are. I also think it’s a responsibility, you know. It’s kind of a commitment, and you feel responsible for your Peer Buddies. You get involved with them. So it’s very rewarding.” – PA6*	

#### Theme I: creating interpersonal connections through opening-up, trust-building, and listening

PAs felt a strong connection to their buddies in having a shared experience. The PAs perceived Peers LEAD as an opportunity for them to connect with their buddies and listen to the buddies share their own experiences of living with diabetes.

*“I would have to say the contact, speaking with them [Peer Buddies], letting them share their journey. That’s what was really beneficial to me” – PA2.*

#### Theme II: opportunities for growth while being a peer ambassador

Peer Ambassadors benefited from the growth they experienced in the Peers LEAD program. They identified how the program helped them contribute to participants’ experiences, adapt to their roles as Peer Ambassadors, and self-reflect on their own journey of living with and managing their type 2 diabetes.

#### Subtheme 1: opportunities to contribute to the program regardless of professional training

Some Peer Ambassadors who were also nurses shared that regardless of their professional training, being a Peer Ambassador was an opportunity for them to learn from others in the program.

*“Beneficial, I would have to say the contact, speaking with them, letting them share their journey. That’s what was really beneficial to me, and I agreed with [PA 1] I didn’t have to be a nurse.” – PA7.*

#### Subtheme 2: opportunities to adapt to the peer ambassador role

Peer Ambassadors shared about their experience with their buddy which in some instances started with conflicts. However, by participating in the program, Peer Ambassadors reflected upon how those conflicts led them to open up more and share about their own frustrating challenges of living with diabetes. By adapting to their role and providing support they benefited in building meaningful relationships.

*“But as time went on, I had a peer buddy who actually was, we just kept bumping heads. It just was not working. But as time went on, the peer buddy I had this time, was like a whole 360. You know, personally, myself, I opened more, and I’ve shared a lot, and I received a lot.” – PA12.*

#### Subtheme 3: opportunities for self-awareness and self-reflection on the diabetes journey

Peer Ambassadors shared how the program allowed them to reflect on their own journey of living with diabetes.

*“I’m just going to say that being a Peer Ambassador helped me to think about how many years I’ve actually been a diabetic. And beginning when things were not*. *.. that first year, and I worked, you know, to be where I am right now. So it’s been an experience. It’s just been an experience. It’s something different, and I’ve enjoyed it.” – PA7.*

### The challenges of being a peer ambassador

Peer Ambassadors described several challenges of providing peer support as: 1) Seeing Peer Buddies experience complications associated with diabetes, 2) Buddies want to share the burden of their family member’s experiences with diabetes, 3) Coming to terms with the responsibilities of being a Peer Ambassador and, 4) Building a relationship and sense of trust with their buddies.

#### Theme I: seeing buddies experience complications associated with diabetes

Some Peer Ambassadors described that it was difficult to hear their PBs describe the medical effects associated with their poorly controlled diabetes.

*“You know, it was hard to see people suffer. It’s difficult to see, I mean, to see how some people have such, both of my Buddies have suffered. They were ill, very ill. Like one is post-stroke. Another one, she has lung disease along with her diabetes, and they’re young. And it was just hard for me to see such guilt in people of that age.” – PA6.*

Peer Ambassadors shared in detail the diabetes-related complications their buddies were experiencing. The Peer Ambassadors were concerned about their buddy’s blood glucose level and poor management of diabetes.

*“And then my Peer Buddy, he had amputated legs, and he has cancer. “– PA8.*

*“You know, to me, it’s unfathomable as far as how one could allow themselves, I’ve heard people say that they had an A1C of 15. I just can’t, for me, that’s hard for me to imagine. You know, I’m not saying that it’s not, I know it’s possible. You know, just to hear that someone has allowed themselves to get to that level, for whatever reason.” – PA8*

#### Theme II: buddies wanted to share the burden of their family members’ experiences with diabetes

Peer Ambassadors expressed the uneasiness they felt after their buddy described the complications associated with a family member’s uncontrolled diabetes.

*“He has a sister that was struggling with a lot of things, and a lot of times he would want to talk about his sister, and the things that she was going through, because she likewise was a diabetic and had gangrene. You just wouldn’t think that people would allow themselves to get to a certain level, but it happens. That was. .. hard, just hearing some of the stories.” – PA8.*

#### Theme III: coming to terms with the responsibilities of being a peer ambassador

Participants mentioned that adjusting to the role of a Peer Ambassador was more difficult than they had anticipated.

*“It was, in the very beginning when I started doing this [Being a Peer Ambassador], it was really difficult for me to start asking questions or saying stuff, because I would feel that I’m saying something stupid or something didn’t make sense. – PA7.*

#### Theme IV: building a relationship and sense of trust with their buddies

Another PA found that forging a bond with their buddy was challenging at first, but it provided them with an opportunity to cultivate a meaningful relationship.

*“I feel, for me, the hardest part was the first couple of conversations with my peer buddy, to get him to buy in to trusting me. And then after he did that, it just took off from there. But getting him to put trust in what we were talking about that I would keep the confidentiality, and we went from there.” – PA3.*

### Rationale for being a peer ambassador

The Peer Ambassadors described their rationale for participating in the Peers LEAD program. These reasons ranged from their desire to receive support for their diabetes self-management as well as to contribute to their communities and to reduce the stigma associated with diabetes. Table 3 illustrates sample quotes from the Peer Ambassadors alongside the identified themes.

**Table 3:** Rationale for being a Peer Ambassador

Themes	Sample quotes
To educate oneself in order to help others	“So, you know, so telling them it’s an experience where you also get to learn stuff, and you also get to tell other people about it. And you’re going to learn a lot of stuff from your peer buddies too. You learn some things from them that you probably didn’t know, or maybe you heard about it, but you forgot about it.” – PA8 “I was a peer ambassador last time, and diabetes is prevalent in my family, and so I wanted to be, wanted to increase my knowledge and speak with others to see what they might be doing and how it might help my family as well as help the community at large.” – PA6
To contribute to decreasing diabetes-related stigma	“If you look at the African American community, you look at a very secretive world. We’re very secret about things, which means we was told not to talk about it. So now we have to try to get these people to actually come in and open up and talk to us about it, you know. And that’s, in itself, you have to be able to do that in order to be a peer ambassador. It’s work that is included in that, you know, and you have to work at being able to, be able to open up with especially older people, to be able to open up to your... African community, totally different. You don’t know who walking around here with diabetes because it’s a hush-hush thing. We don’t talk about it. We don’t...” – PA8 “I saw a need in our community. People that, of color, primarily black, who are having difficulty opening up, asking questions.” – PA7
Provides the opportunity to receive support for one’s diabetes experience	This is really the first time I’ve actually talked with other people, other than the doctor, about the diabetes. Sometimes people can just deal with it by themselves, and as everybody here knows, there are times you feel encouraged and times you feel discouraged, because the numbers don’t look right, or, and it’s a marathon. It’s like a long-distance race, and it helps to be able to talk to somebody, yeah.” – PA4 “I’m a nurse and because I’m a diabetic myself, I felt that I could use the support myself also.” – PA1

#### Theme I: to educate oneself in order to help others

Most of the participants agreed that being a Peer Ambassador allowed them to educate themselves about their diabetes in order to help their Peer Buddies, their family members, and/or their communities struggling with diabetes management. Several Peer Ambassadors highlighted that they wanted to gain personal knowledge about diabetes prior to assisting others with the disease.

*“The reason why I wanted to become an ambassador is, because, I wanted to educate myself on diabetes, and then, at the same time, be able to reach out and help someone in the community.” – PA8.*

#### Theme II: to contribute to decreasing diabetes-related stigma

Some Peer Ambassadors described the lack of dialogue about diabetes in the African American community and their desire to make a difference.

*The reason I wanted to be an ambassador was because when I was approached about it. .. diabetes, something that was not exactly talked about in our community. So, therefore, I wanted to learn more, and I wanted to give out more to other people, be able to give it out to other peoples who have diabetes. Then they don’t have to be ashamed of it, and that, you know, we can talk freely about it. I wanted to be so that we can just talk so freely about it, you know, instead of hiding it.” – PA10.*

#### Theme III: provides the opportunity to receive support for one’s diabetes experience

Other Peer Ambassadors indicated that they wanted to share their experiences and offer support to others with diabetes in a safe and welcoming environment. Similarly, to learn more about their diabetes, Peer Ambassadors saw their role as an opportunity for them to personally open up about their own experiences and receive the support they also needed.

Peer Ambassador who were nurses, identified how their role as Peer Ambassador differed from their profession and gave them the opportunity to receive support.

*“I am a diabetic. I’m a nurse as well. And the reason why I agreed to do it is because I think that a lot of people, they have a lot of questions and a lot of concern. And I thought that maybe I could answer some of them on a personal level, you know, share my experiences, and let them know that they’re not alone. We all struggled in the beginning to get hold. I didn’t always have perfect A1Cs either, you know, so that’s why I decided to do it.” – PA7.*

## Discussion

The study results showed that peer supporters in diabetes self-management programs aim to provide encouragement for other people with diabetes and to receive support for themselves. They view providing peer support as a responsibility, and they also benefit from their peer support role. These benefits include creating interpersonal connections, experiencing opportunities for growth through self-awareness and self-reflection, and finding reward in the commitment that comes with supporting others. Notwithstanding the benefits, African Americans described some challenges associated with peer support, especially when supporting peers who were seriously ill or who struggled with medication management despite the education provided during the program. One initial challenge for some of peers was creating a trusting relationship. Others struggled with accepting the terms of their roles as trained supporters. Nonetheless, the reasons African Americans partake in peer support consist of educating themselves to help others, decreasing diabetes-related stigma in African American communities, and receiving support themselves.

Our study adds to the existing literature on peer support in several ways. First, our study adds a new dimension to the benefits of providing peer support. Prior studies of peer support only emphasize the clinical benefits of the programs to the peers involved [9, 30, 31]. Analogously, a study of key features of peer support in chronic disease management revealed that people participating in peer support interventions only express interest in receiving information and answers to their questions, rather than gaining emotional support [32]. However, when African Americans shared the benefits of the Peers LEAD program they focused on the social and emotional benefits of the program. They mentioned how the program helped them grow, open up and its impact the program had on their relationships with their peers. They also mentioned the reward they feel because of their commitment to their role. Other studies exploring peer support should consider examining the psychosocial benefits of the programs to the participants and the impacts these benefits may have on other outcomes of interest.

Second, existing literature recognizes peer support as a mutually beneficial relationship where individuals give and receive support from each other [33]. Our Peer Ambassadors reflected this finding by recognizing the benefits of peer support as an experience that enlightens their self-awareness and reflection on their journeys of living with diabetes. One of the benefits of peer support for African Americans is forming meaningful connections with their peers through trust-building. Trust is significantly associated with participant retention and peer support programs are recommended to include as strategies to enhance trust within their interventions [34].

Third, trust has been associated with reasons why African Americans living with type 2 diabetes do not seek out support from their peers. A study revealed that African Americans were distrustful about disclosing their type 2 diabetes status to their peers and seeking out support [35]. One of the peers in our study described the African American community as “a very secretive world.” Therefore, the Peer Ambassador joined the Peers LEAD program to contribute to decreasing diabetes-related stigma in African American communities. Other Peer Ambassadors recognized that African Americans have difficulties opening up about their diabetes. A Peer Ambassador identified that the most challenging part of the Peers LEAD program was the first conversations they had with their buddies before building trust. Another participant revealed that our Peers LEAD program is the first time they had spoken to anyone other than their physician about their diabetes. The reluctance to be vulnerable with peers has been identified in one study as relating to the stigma attached to type 2 diabetes in African American communities [35]. Effective peer support programs must respect and acknowledge the needs of the person with diabetes and take into consideration privacy concerns while building trust with peers to ultimately embolden them to open up about their diabetes.

Familial relationships are ingrained in cultural beliefs that impact medication adherence among African Americans [36]. For African Americans who had concerns about trust, they felt that they depend upon their family and friends for support. In another study, African Americans revealed that their impression after their type 2 diabetes diagnosis was that their families would be their primary support system [35]. Another study sought input from African American pastors and found that families carry the burden of caring for someone with diabetes [37]. Therefore, it is not surprising that African Americans in our study shared their challenges in supporting peers who were focused on the burden of caring for family members with type 2 diabetes. Due to the high prevalence of diabetes in African American communities and the burden shared with family members, there is a possibility that during peer support programs peers may want to discuss their family members’ experiences with diabetes. One Peer Ambassador discussed their rationale for joining the program as an opportunity to become more knowledgeable about diabetes. Current literature suggests that diabetes self-management interventions have shifted from a didactic knowledge approach to a self-management and empowerment-centered approach [38]. Disease self-management education allows for an ongoing facilitation of knowledge needed to manage diabetes [39]. Empowerment is the process through which people gain control over decisions and actions affecting health [40]. People are empowered when they have adequate knowledge about their diabetes [40, 41]. Peer support interventions should continue to focus on educating those providing peer support about diabetes self-management.

Our Peer Ambassadors discussed how the knowledge they gained about their diabetes is to help themselves as well as their families and their communities. Individuals’ rationale for participating in a peer support program to help their communities can be further explored especially in under-resourced communities. Studies evaluating the benefits of diabetes self-management programs to individuals and their families show that families may benefit directly from attending health education programs [42, 43]. However, the impact of diabetes peer support programs beyond families, towards a focus on communities are yet to be explored. Future studies can evaluate how those providing peer support extend their support to their communities and the impact of the support on the health of their communities.

Peer Ambassadors had difficulties seeing their peers suffer from diabetes-related complications. Peer support programs can provide emotional support to peers in their roles of supporting individuals struggling with type 2 diabetes. Other studies can further explore in-depth the challenges peers face when providing support and design interventions to equip them with the necessary tools to cope and be efficient in their roles. These roles do not require professional expertise, and although two peers in our study identified as nurses, their professional training was not necessary to provide peer support.

While African Americans experience challenges in their roles as peer ambassadors, they participate in peer support programs to benefit themselves and their communities. A study of peer support in low-income African Americans found that sharing illness experiences in the group setting gave participants practical ideas on how to manage their illness [44]. Peer Ambassadors spoke about the growth they experience in opening up, building trusting relationships, and learning about diabetes to help themselves and their communities. Our study offers a basis for further exploration of the experiences of African Americans providing peer support. It is evident that the benefits of providing peer support are not limited to the individual receiving the support, rather, it is a mutually beneficial relationship.

## Limitation

There are several limitations to our study. Focus groups have the advantage of eliciting the views, feelings, and perceptions of a group [45, 46]. However, participants who share different views may not be comfortable sharing them in a focus group.

The study findings were based on perceptions of middle-aged African American adults with diabetes in one Midwestern state. Experiences of ambassadors providing peer support might differ among age groups and regional locations. Future studies should consider exploring these experiences among other age groups of African Americans in different geographic locations. It is important to note that the sample size was 12 participants, which is consistent with the acceptable sample size in qualitative research for focus groups. However, two Peer Ambassadors that participated in the program did not participate in the focus group due to time constraints. Despite these limitations, the findings of this study can inform future research, as well as peer support training and programs.

## Conclusion

As the number of people with diabetes grows exponentially, particularly in low-resource communities, the evidence continues to grow for peer support as a viable and compelling approach to lifelong diabetes self-management. African Americans providing peer support perceive their roles as beneficial to them and their peers. The challenges they experience in their roles are linked to the emotional difficulties of seeing peers experience diabetes-related complications. Peer support programs in African American communities should consider the benefits and rationale for participants’ involvement in their programs and address the challenges they face when providing support.

## Data Availability

The dataset used and/or analyzed for the current study are available from the corresponding author on reasonable request.

## Abbreviations

PA: Peer Ambassador
PB: Peer Buddy
Peers LEAD: Peers Supporting Health Literacy, Self-Efficacy, Self-Advocacy, and Adherence
